# Chromogenic culture media complements diagnostic cytology in the visual identification of pathogenic skin bacteria in dogs and cats

**DOI:** 10.3389/fvets.2023.1152229

**Published:** 2023-07-11

**Authors:** Miha Avberšek, Julian Ihssen, Greta Faccio, Urs Spitz, Blaž Cugmas

**Affiliations:** ^1^Veterinary Clinic Zamba, Vets4science d.o.o., Celje, Slovenia; ^2^Biosynth AG, Staad, Switzerland; ^3^Institute of Atomic Physics and Spectroscopy, University of Latvia, Riga, Latvia

**Keywords:** chromogenic medium, chromogenic agar, skin pathogens, pyoderma, otitis externa, point-of-care assay, bacterial identification, veterinary dermatology

## Abstract

In dogs and cats, bacterial skin infections (pyoderma and otitis externa) are a common cause for visiting the veterinary clinic. The most frequent skin pathogens are *Staphylococcus pseudintermedius*, *Staphylococcus aureus*, *Escherichia coli*, and *Pseudomonas aeruginosa*, often requiring different therapeutic antibiotic protocols. Unfavorably, existing diagnostics based on cytology cannot reveal bacterial species but only bacterial shapes such as cocci or rods. This microscopic limitation could be overcome by clinical translation of affordable chromogenic media, which enable species identification based on bacterial colonies growing in different colors and sizes. In this study, we determined how well inexperienced general veterinary clinicians identified bacterial pathogens from the skin and ears on two commercial (Chromatic™ MH and Flexicult® Vet) and one custom-made Mueller Hinton agar-based chromogenic medium. For this purpose, four veterinarians evaluated 100 unique samples representing 10 bacterial species. On average, clinicians correctly identified between 72.1 and 86.3% of bacterial species. Colony colors developed quickly on the Chromatic™ MH medium, leading to the highest 81.6% identification accuracy after 24 h incubation. However, Flexicult® Vet exhibited the highest accuracy of 86.3% after prolonged 48 h incubation. Evaluators easily recognized bacteria displaying uniquely colored colonies like green-brown *Pseudomonas aeruginosa*, blue *Enterococcus faecalis*, orange-brown *Proteus* spp., and red *Escherichia coli*. Oppositely, staphylococci shared uncharacteristically pale pink colonies causing misidentifications among the genus, deteriorating overall accuracy by around 10 percentage points (from 90.9%). Another reason for identification errors was the evaluators’ inexperience, reflected in not recognizing colony size differences. For example, although *Streptococcus canis* exhibited the tiniest colonies, the species was frequently mistaken for other cocci. Finally, around 10% of errors were negligence-related slips due to unconsidered sample history. To conclude, the introduction of chromogenic media into veterinary clinics can significantly complement diagnostics in skin inflammations by identifying pathogen species in around 80% of cases. The extra information may help in therapeutic dilemmas on antibiotics and standard antimicrobial susceptibility testing. Additional personnel training and evaluation help by visuals, flowcharts, checklists, and, if necessary, microbiologists could further improve identification accuracy.

## 1. Introduction

Skin diseases in dogs and cats are one of the main reasons for visiting a veterinarian. Bacterial or fungal infection of the skin (pyoderma) and external ear canal (otitis externa), parasitic infestation, and neoplasia account for the majority of dermatological cases (1, 2). *Staphylococcus pseudintermedius* (*S. pseudintermedius*) causes most pyodermas in dogs (3–5) and *S. aureus* in cats (6–9). Other less common infection-causing bacteria are *Staphylococcus felis* (*S. felis*) (in cats), *Staphylococcus schleiferi* (*S. schleiferi*), *Escherichia coli* (*E. coli*), *Enterococcus faecalis* (*E. faecalis*), *Klebsiella* spp., *Proteus* spp., *Pseudomonas aeruginosa* (*P. aeruginosa*), *Corynebacterium* spp., and *Streptococcus canis* (*S. canis*) (4, 7–11). The scientific and medical community pays special attention to methicillin-resistant *S. pseudintermedius* (MRSP) and *S. aureus* (MRSA) due to their potential impact on the health of animals and people (12). More troublesome, Blondeau et al. (13) demonstrated that *S. pseudintermedius* directly transmitted from a family dog caused a urinary tract infection in a human. Finally, bacteria such as *P. aeruginosa* may produce biofilms leading to very challenging treatment of (often persistent) otitis externa (14).

Diagnosing skin and ear infections relies on cost-effective and fast cytology (15). A clinician swabs the inflamed spot and stains the deposited material on the microscopic slide, generally with commercial Romanowsky stains like Diff-Quik™ (RAL Diff-Quik™, CellaVision, Lund, Sweden). Observation by microscope can reveal inflammatory response cells (e.g., neutrophils) and pathogens like bacteria or yeasts (e.g., *Malassezia pachydermatis*). A significant cytological shortcoming is that bacterial identification is limited to their shape, i.e., round cocci (*Staphylococcus* spp., *Enterococcus* spp.) and elongated rods (*E. coli*, *P. aeruginosa*). Therefore, cytology does not significantly contribute to our awareness of potentially present hazardous strains of bacteria (1, 16).

The limited availability of affordable and straightforward diagnostic tools probably contributes to the fact that most antibiotics are still empirically prescribed (17). Moreover, standard microbiological analysis with bacterial culture and antimicrobial susceptibility testing (AST) is rarely employed. Culture and AST are mostly reserved for complicated and persistent inflammations (17, 18), although being recommended for diagnosing pyodermas that require systemic antibiotic therapy (1, 12, 15, 19). The standard microbiological analysis exhibits a few days-long (>3) turnaround time, and its costs often exceed the expenses for antibiotic therapy (10, 12, 17).

**Figure 1:**
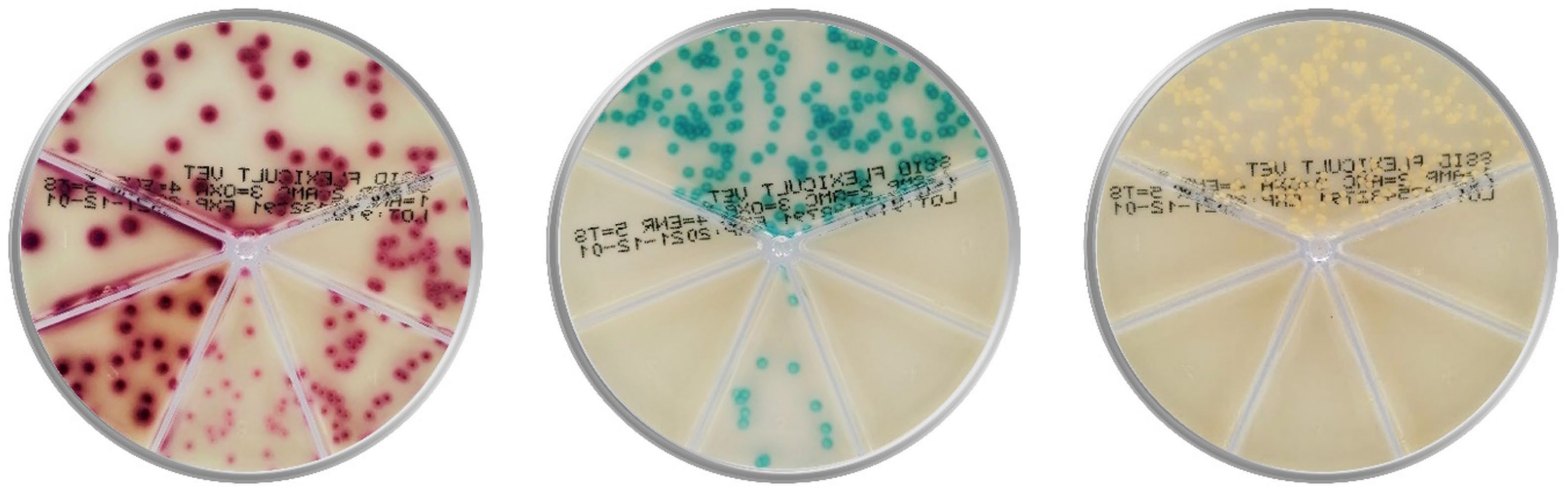
Culture of *Escherichia coli* (on the left)*, Enterococcus faecalis* (in the middle), and *Staphylococcus aureus* (on the right) on the popular chromogenic media Flexicult® Vet (SSI Diagnostica, Hillerød, Denmark), offering primary bacterial identification and antimicrobial susceptibility testing in canine and feline urinary tract infections (Agar plate images are acquired in the PetriView BOX, Vets4science, Celje, Slovenia).

Consequently, a few affordable and faster point-of-care (POC) tests appeared to complement cytology and offer bacterial identification and AST. These tests are expected to improve veterinary adherence to the consensus antibiotic guidelines and increase the use of standard microbiological analysis (17). Two well-known POC tests are chromogenic culture media-based Flexicult® Vet (Figure 1, SSI Diagnostica, Hillerød, Denmark) and Speed Biogram (Virbac, Carros, France). Identification and AST are based on the color and presence of bacterial growth (20–22).

However, chromogenic culture media accuracies seemed inconsistent since the reported Flexicult® Vet values for urinary pathogen identification and AST ranged between 39 and 100% (20, 21, 23, 24). Enrolled experts (microbiologists and microbiological technicians) outperformed veterinary clinicians in bacterial identification by 16 (68.7 and 84.6%) (21) and 47 percentage points (53 and 100%) (20), indicating the correlation between medium performance and evaluator’s experience. Another essential factor in POC test performance could be the selection of enzyme substrates, releasing specific signalophors after the enzymatic reaction (25). In these media, chromophores ensure that bacterial colonies (or agar) exhibit particular colors (Figure 1). Undoubtedly, the differentiation of several bacteria on the same plate often requires a combination of substrates optimized for the anticipated pathogens.

Existing studies primarily focused on identifying urinary bacteria by a single, mostly experienced evaluator using a specific chromogenic culture medium. Oppositely, we wanted to determine how well general clinical veterinarians identified bacterial pathogens from the skin and ears on several chromogenic media. Two enrolled agars were available on the market and adjusted for general or urinary pathogens. The last medium was custom-made with commercially available enzyme substrates, focusing on identifying typical skin pathogens like distinguishing *Staphylococcus* species between themselves and from other bacteria. Successful identification of bacterial species on affordable chromogenic culture media could complement cytology, helping the clinician to select proper antibiotics, conduct in-clinic agar-based AST, or decide whether further diagnostic tests are needed.

## 2. Materials and methods

The ethics committee (Ministry of Agriculture, Forestry and Food, Republic of Slovenia) granted the ethical acceptability of this study (No. U34405-246/2022/18, Administration for Food Safety, Veterinary Sector, and Plant Protection, Republic of Slovenia).

### 2.1. Bacterial samples

We collected 36 bacterial isolates from pyoderma and otitis externa in dogs and cats visiting our veterinary clinic (Table 1). Bacterial species were determined by a certified microbiological laboratory (University of Ljubljana, Ljubljana, Slovenia) utilizing matrix-assisted laser desorption/ionization time-of-flight (MALDI-TOF) mass spectrometry (Microflex LT system, Bruker Daltonics, Bremen, Germany). The accumulated isolates representing 10 bacterial species served to prepare 100 agar plates in different bacterial concentrations between 200 and 2000 colony-forming units (CFU) per plate. The number of prepared samples per species was based on the typical pathogen prevalence while ensuring that at least five samples represented each species (Table 1).

**Table 1:** The number of clinically collected bacterial strains and prepared samples (i.e., agar plates).

Bacterial species	No. of unique strains	No. of prepared samples
*Staphylococcus pseudintermedius*	13	25
*Staphylococcus aureus*	2	8
*Staphylococcus felis*	3	10
*Staphylococcus schleiferi*	1	5
*Streptococcus canis*	2	9
*Escherichia coli*	4	10
*Enterococcus faecalis*	4	10
*Pseudomonas aeruginosa*	3	10
*Proteus* spp.	2	8
*Corynebacterium auriscanis*	2	5
Total	36	100

### 2.2. Chromogenic media

We studied three Mueller Hinton agar-based chromogenic media. Two enrolled agars were found on the market, while the third medium was custom-made with commercially available enzyme substrates.

*LFCA*: Liofilchem Chromatic™ MH (Liofilchem, Roseto degli Abruzzi, Italy). We prepared the plates from the supplied powder according to the instructions.

*FLEX*: Flexicult® Vet Scandinavia (SSI Diagnostica, Hillerød, Denmark). We bought agar plates as ready-to-use.

*CAVD*: Custom-made chromogenic medium based on the Mueller Hinton II agar (Merck Millipore, Tullagreen, Ireland). After autoclaving for 15 min at 121°C, the molten agar was mixed by magnetic stirring and cooled to 50°C in a water bath. Then, 100 mM stock solutions of chromogenic substrates in dimethyl sulfoxide (DMSO) and 0.5 M stock solutions of isopropyl-beta-D-thiogalactopyranoside (IPTG) and 1-O-Methyl-beta-D-glucuronic acid, sodium salt in ultrapure water were prepared and sterilized by filtration. IPTG is an inducer for beta-galactosidase and 1-O-Methyl-beta-D-glucuronic acid is an inducer for β-glucuronidase. After adding chromogenic substrate and inducer stock solutions (1 to 4 ml per liter, depending on the final concentration), the molten agar was poured into sterile single-use plastic Petri dishes and left to solidify. Plates were stored in a refrigerator in plastic bags to prevent drying. The final concentrations of chromogenic substrates were: 0.15 mM Aldol® 467 beta-D-galactopyranoside (product number A-4676, Biosynth AG, Staad, Switzerland), 0.14 mM 5-Bromo-4-chloro-3-indoxyl-beta-D-glucuronic acid, sodium salt trihydrate (product number B-7400, Biosynth AG), 0.12 mM 5-Bromo-6-chloro-3-indoxyl phosphate, disodium salt trihydrate (product number B-7453, Biosynth AG), 0.2 mM 5-Bromo-4-chloro-3-indoxyl-beta-D-glucopyranoside (product number B-7250, Biosynth AG), 0.5 mM IPTG, 0.5 mM 1-O-Methyl-beta-D-glucuronic acid, sodium salt (product number M-3600, Biosynth AG), and 0.35% DMSO.

### 2.3. Sample preparation

We inoculated Mueller Hinton liquid medium (Biokar, Allonne, France) with known bacterial species (Table 1). After 24 h of incubation at 37°C, we prepared dilutions of bacterial suspension in distilled water to cultivate between 200 and 2000 distinct CFU on each plate. Then, we transferred 100 μl of the suspension onto the LFCA and CAVD agars and smeared it in three directions with a sterile inoculation loop. According to the manual, 1 ml of suspension was added to the entire surface of Flexicult® Vet plates, and the excess fluid was decanted. In total, we inoculated 300 plates (100 per medium), which were incubated for 48 h at 37°C.

### 2.4. Evaluations

After 24 and 48 h of incubation, agar plates were evaluated visually by three small and one large animal clinical veterinarians, all without experience in microbiological diagnostics. Due to the numerous plates (300) and evaluations (1800), we divided the study into four sessions with 30–90 plates per session. A function *randperm* (Matlab R2016a, MathWorks, Natick, USA) ensured a random sample (count) selection. Three evaluators participated in each evaluation session, performing 432 (Evaluator 1), 600 (Evaluator 2), 408 (Evaluator 3), and 360 evaluations (Evaluator 4). Evaluators examined all samples on one medium before addressing the next one. The medium order was random and unique for each session (function *randperm*, Matlab, MathWorks). As an evaluation result, the evaluator selected one of the 10 possible bacteria species for each agar plate. From the following analysis, we removed 12 evaluations since three agars with *S. canis* (two LFCAs at 24 h and one CAVD at 24 and 48 h) did not exhibit any growth.

**Figure 2:**
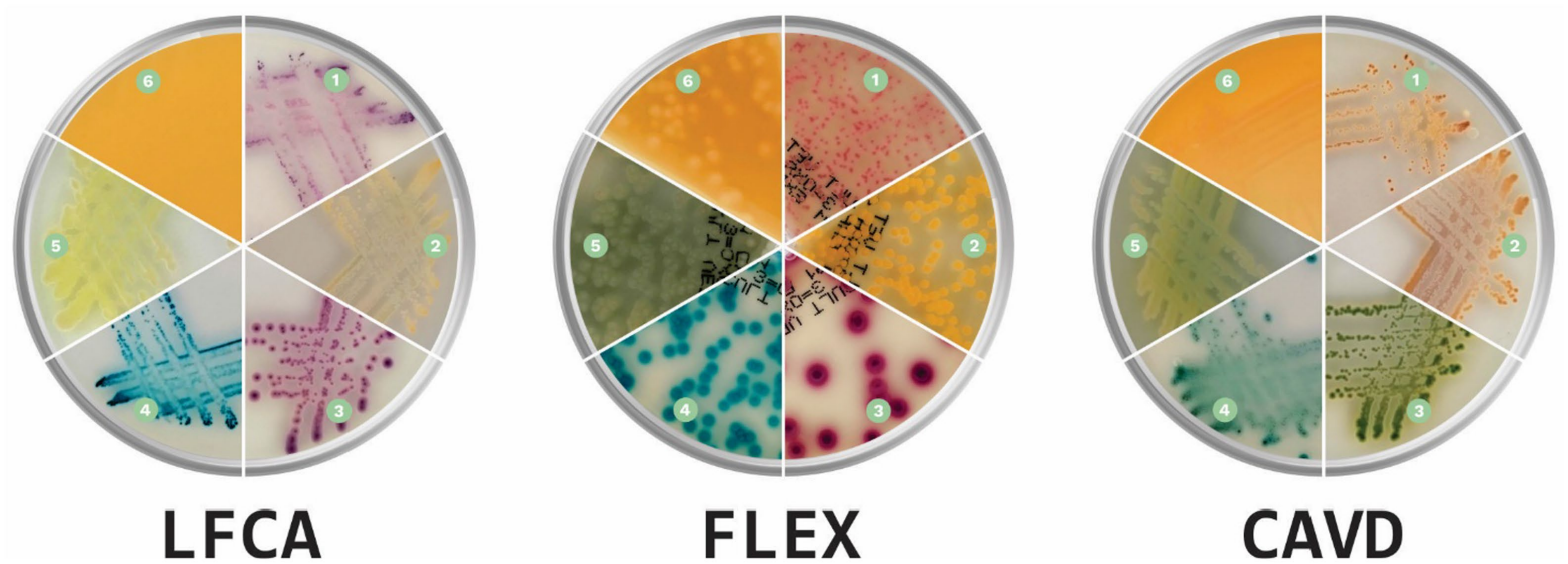
Prevalent canine and feline skin bacteria (1) *Staphylococcus pseudintermedius*, (2) *Staphylococcus aureus*, (3) *Escherichia coli*, (4) *Enterococcus faecalis*, (5) *Pseudomonas aeruginosa*, and (6) *Proteus* spp. on three chromogenic media: Liofilchem Chromatic™ MH (LFCA), Flexicult® Vet Scandinavia (FLEX), and a custom-made chromogenic agar (CAVD; Agar images were acquired in the PetriView BOX, Vets4science, Celje, Slovenia).

Before evaluations, veterinarians were briefly introduced to each medium’s characteristics, the appearance of colors specific to each bacterium, and the morphological characteristics of the colonies (Figure 2). Evaluators had instant digital access to a manual with bacterial images on all three media during the whole duration of the study. Additionally, we supplemented each sample with information on the sample history: *animal* (dog or cat), *sample location* (skin or ear), and *microscopic pathogen shape* (cocci or rods). Due to the collected samples and for the sake of study simplicity, we informed evaluators that *S. felis* (cats) and *S. pseudintermedius* (dogs) samples are animal-specific.

### 2.5. Statistics

We calculated accuracies, agreements, sensitivities, and specificities in the programming language Matlab (MathWorks). We defined accuracy as the ratio between correct bacteria identifications against the gold standard (MALDI-TOF) and all samples. Agreements with the gold standard and between evaluators (inter-rater) were estimated by Krippendorff’s Alpha (*α_K_*). We evaluated the significant differences between the agreements with a non-parametric Mann–Whitney U test. The sensitivity for each pathogen was calculated as a ratio between true positives (correctly identified) and the number of correct identification at the gold standard. Oppositely, specificity was a ratio between true and all negatives (i.e., identifications of other bacterial species at the gold standard). In the manuscript, *error* (also *misidentification*) stands for all wrong bacterial species identifications, including false positives and false negatives.

## 3. Results

All three enrolled chromogenic media performed similarly, exhibiting the mean identification accuracy (a percentage of correctly identified bacteria) between 72.1 and 86.3% (Table 2). Prolonged incubation improved bacterial identification, and consequently, the mean accuracy increased by 5.7 percentage points, from 76.4% (24 h) to 82.1% (48 h). The difference was statistically significant (*p* = 0.049, Mann–Whitney *U* test, function *signrank*, Matlab). Only LFCA agar, studied by evaluator 3, performed worse with additional incubation time (90.9 vs. 82.8%). Evaluators 2 and 3 reached high, ~90.0% accuracy using LFCA and FLEX media. On the other hand, evaluator 4 consistently delivered the worst performance, once recognizing only 62.1% bacterial species (LFCA medium).

**Table 2:** Mean accuracies in bacterial identification (%) by all (Accuracy) or a single evaluator after 24 and 48 h incubation on average (All) or per specific agar: Liofilchem Chromatic™ MH (LFCA), Flexicult® Vet Scandinavia (FLEX), and custom-made agar (CAVD).

	24 h incubation				48 h incubation			
	All	LFCA	FLEX	CAVD	All	LFCA	FLEX	CAVD
Accuracy	76.4 ± 9.9	81.6 ± 12.8	75.7 ± 10.6	72.1 ± 7.0	82.1 ± 4.8	82.7 ± 8.5	86.3 ± 3.5	77.1 ± 3.5
Evaluator 1	81.0	86.1	81.9	75.0	81.5	84.7	84.7	75.0
Evaluator 2	75.1	83.7	71.0	70.7	86.3	90.0	90.0	78.8
Evaluator 3	85.6	90.9	86.8	79.1	82.8	80.9	86.8	80.6
Evaluator 4	62.7	62.1	63.3	62.7	74.9	70.0	81.7	72.9
*Staph.* spp. grouped	86.9 ± 9.8	90.1 ± 9.4	85.7 ± 13.4	84.9 ± 6.7	90.9 ± 3.5	88.0 ± 5.3	94.7 ± 2.0	89.9 ± 4.3

Accuracies are also listed when all *Staphylococcus* species are grouped in the same species category (*Staph*. spp. grouped). Values listed after “±” mark standard deviation.

Staphylococci exhibit similar colors on chromogenic media; thus, we also studied accuracies when all *Staphylococcus* samples were grouped in the same species category. In this case, identification accuracy improved, on average, by 8.8–10.5 percentage points (Table 2).

Similar to the accuracies, agreement in bacterial identification between chromogenic agars and the gold standard (i.e., MALDI-TOF) improved with prolonged incubation, i.e., Krippendorff’s Alpha (*α_K_*) increased from 0.73 to 0.79 (Table 3, *p* = 0.092, Mann–Whitney *U* test). Surprisingly, inter-rater reliability (IRR, i.e., agreement between evaluators) was in the same range as the agreement with the gold standard.

**Table 3:** Mean agreement (Krippendorff’s Alpha *α_K_*) and (±) standard deviation between the gold standard (MALDI-TOF) and evaluators, and between evaluators (Inter-rater reliability) in bacterial identification after 24 and 48 h incubation on average (All) or per specific agar: Liofilchem Chromatic™ MH (LFCA), Flexicult® Vet Scandinavia (FLEX), and custom-made agar (CAVD).

	24 h incubation				48 h incubation			
	All	LFCA	FLEX	CAVD	All	LFCA	FLEX	CAVD
Agreement (*α_K_*)	0.73 ± 0.11	0.79 ± 0.15	0.72 ± 0.12	0.67 ± 0.08	0.79 ± 0.05	0.80 ± 0.10	0.84 ± 0.04	0.73 ± 0.04
Inter-rater reliability	0.69 ± 0.12	0.72 ± 0.08	0.64 ± 0.20	0.71 ± 0.18	0.80 ± 0.06	0.74 ± 0.08	0.84 ± 0.05	0.82 ± 0.11
IRR(best) / IRR(worst)	0.81 / 0.49	0.81 / 0.59	0.88 / 0.42	0.96 / 0.43	0.91 / 0.75	0.85 / 0.64	0.88 / 0.74	1.00 / 0.71

IRR (best) and IRR (worst) mark the best and worst agreement between two evaluators.

Evaluators ideally identified bacterial species that grew in unique colors (Table 4), reaching almost perfect (~100%) sensitivities and specificities for bluish *E. faecalis*, orange-brown *Proteus* spp., green-brown *P. aeruginosa* and red *E. coli*. Oppositely, differentiation between *Staphylococcus* species was challenging due to similar shades of bacterial colonies. For instance, less common *S. schleiferi* was rarely recognized (sensitivity of ~9%). Evaluators satisfactorily identified only *S. pseudintermedius* and *S. felis* (sensitivity and specificity between 81 and 100%).

**Table 4:** Sensitivities and specificities (in %) in identifying separate bacterial species after 24 or 48 h incubation, average on all (All) or specific agar: Liofilchem Chromatic™ MH (LFCA), Flexicult® Vet Scandinavia (FLEX), and custom-made agar (CAVD).

Bacteria	Sensitivity (%)								Specificity (%)							
	24 h incubation				48 h incubation				24 h incubation				48 h incubation			
	All	LFCA	FLEX	CAVD	All	LFCA	FLEX	CAVD	All	LFCA	FLEX	CAVD	All	LFCA	FLEX	CAVD
*S. pseudintermedius*	86.2	100	84.0	74.7	92.4	92.0	100	85.3	89.8	95.0	91.1	83.3	89.6	94.2	92.9	81.5
*S. aureus*	31.9	70.8	20.8	4.2	37.5	54.2	54.2	4.2	97.9	96.7	97.8	99.3	98.1	97.1	97.5	99.6
*S. felis*	81.1	66.7	80.0	96.7	87.8	86.7	80.0	96.7	96.1	97.0	95.9	95.5	97.3	99.3	96.7	95.9
*S. schleiferi*	8.9	20.0	6. 7	0	8.9	26.7	0	0	99.1	99.3	97.9	100	99.9	100	99.7	100
*S. canis*	61.1	71.4	63.0	50.0	65.4	81.5	74.1	37.5	93.4	95.2	92.7	92.3	96.5	96.0	97.4	96.0
*E. coli*	93.3	96.7	96.7	86.7	96.7	93.3	96.7	100	99.8	100	99.6	99.6	99.6	99.3	100	99.6
*E. faecalis*	83.3	60.0	100	90.0	88.9	66.7	100	100	98.9	98.5	98.2	100	98.1	95.6	99.6	99.3
*P. aeruginosa*	85.6	90.0	83.3	83.3	97.8	96.7	100	96.7	99.3	100	99.3	98.5	99.9	99.6	100	100
*Proteus* spp.	94.4	95.8	91.7	95.8	100	100	100	100	99.2	99.3	99.3	98.9	99.8	99.6	100	99.6
*C. auriscanis*	86.7	86.7	73.3	100	88.9	86.7	93.3	86.7	99.7	100	100	98.9	99.8	99.3	100	100

**Figure 3:**
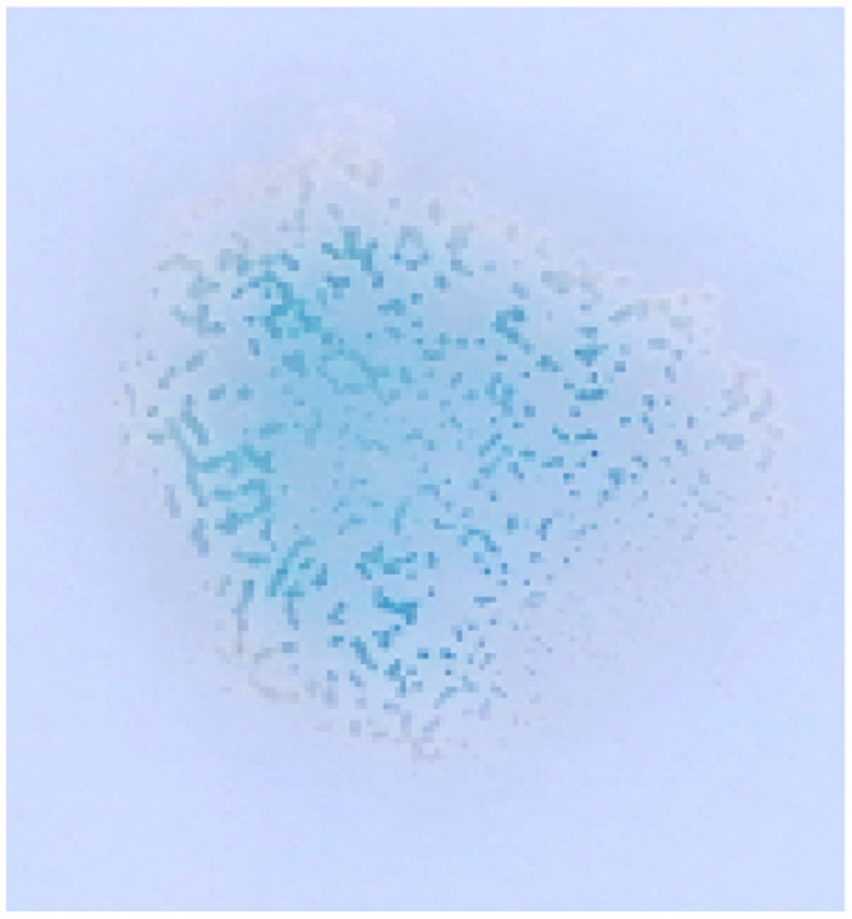
*Staphylococcus aureus* colonies, typically intense yellow, occasionally exhibited blue color after 48 h incubation on an LFCA medium (Liofilchem Chromatic™ MH).

**Figure 4:**
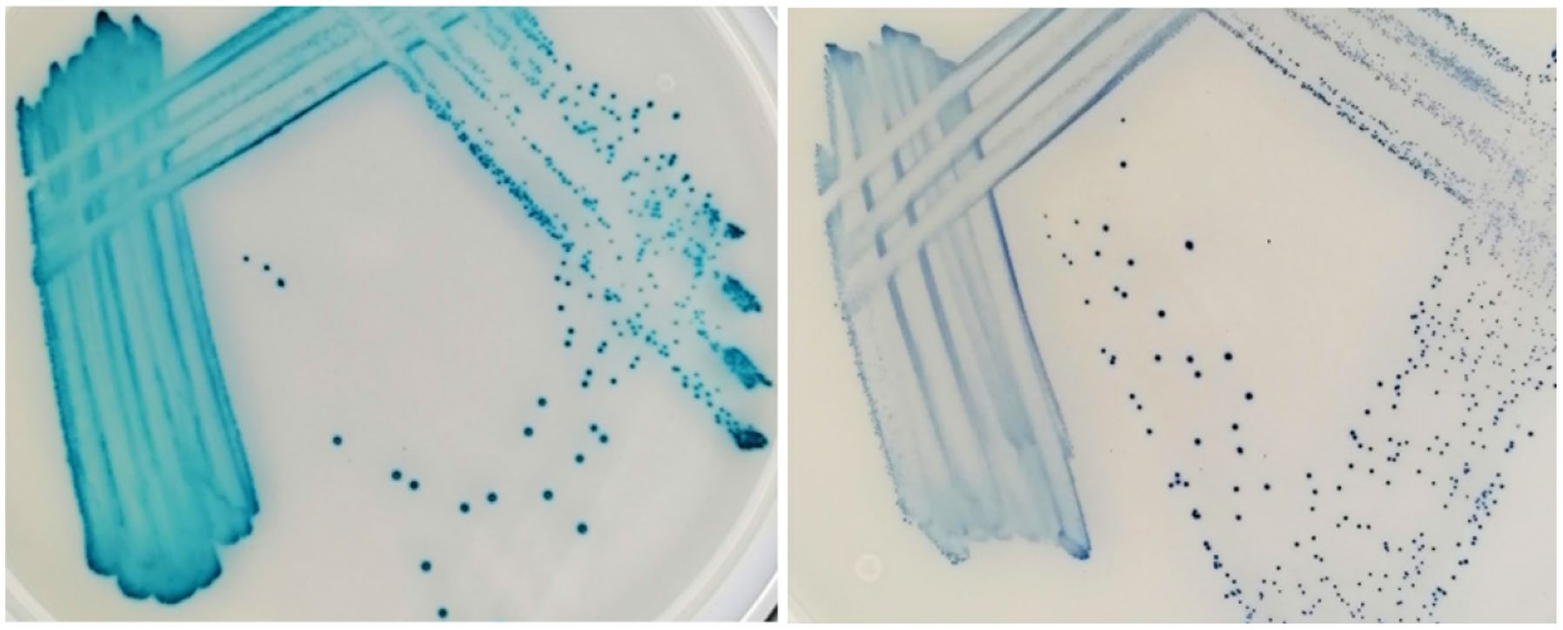
*Enterococcus faecalis* (left) and *Streptococcus canis* (right) after 48 h incubation on the Liofilchem Chromatic™ MH medium plates (LFCA).

For staphylococci and *S. canis*, misidentifications mostly happened within the mentioned bacteria (Table 5). Other notable errors included typically intense yellow-exhibiting *S. aureus*, which sometimes turned bluish after 48 h incubation on LFCA agar (Figure 3) and was consequently identified as *E. faecalis*. Furthermore, *E. faecalis*, after 24 h incubation on LFCA, was occasionally mistaken for *S. canis*, although these bacteria do not exhibit the same colony size (Figure 4). Finally, there were a few misidentifications between *S. pseudintermedius* and *E. coli* due to similar pink-red colony shades on LFCA and FLEX media. There were no errors on CAVD agar since *E. coli* colonies were green (Figure 2). Based on the errors in human healthcare and laboratory medicine (26, 27), we classified errors when interpreting chromogenic agars into three categories related to chromogenic medium or evaluator (knowledge- and negligence-based; Table 6).

**Table 5:** The number of bacteria samples (%) mistaken for another bacteria species (per row) after 24 and 48 h incubation for all (All) or per specific agar: Liofilchem Chromatic™ MH (LFCA), Flexicult® Vet Scandinavia (FLEX), a custom-made agar (CAVD).

		*S. p.*	*S. aureus*	*S. felis*	*S. schleiferi*	*S. canis*	*E. coli*	*E. faecalis*	*P.aeruginosa*	*Proteus* spp.	*C.auriscanis*
		24 h, 48 h	24 h, 48 h	24 h, 48 h	24 h, 48 h	24 h, 48 h	24 h, 48 h	24 h, 48 h	24 h, 48 h	24 h, 48 h	24 h, 48 h
*S. pseudintermedius*	All LFCA FLEX CAVD		9.7, 5.9 -, - 16.7, - **5.3**, 9.1		12.9, - -, - 33.3, - -, -	74.2, 76.5 -, 50.0 41.7, - 94.7, 90.9	3.2, 11.8 -, 33.3 8.3, - -, -		-, 5.9 -, 16.7 -, - -, -		
*S. aureus*	All LFCA FLEX CAVD	40.8, 28.9 28.6, - 21.1, - 60.9, 56.5		40.8, 33.3 57.1, 9.1 42.1, 54.6 34.8, 34.8		18.4, 13.3 14.3, - 36.8, 45.5 4.4, 4.4		-, 24.4 -, 90.9 -, - -, 4.4			
*S. felis* *	All LFCA FLEX CAVD	23.5, 81.1 30.0, 100.0 16.7, 67.7 -, 100.0	17.7, - 20.0, - -, - 100, -		11,8, - 20.0, - -, - -, -	29,4, 18.2 -, - 83.3, 33.3 -,-					
*S. schleiferi*	All LFCA FLEX CAVD	48.8, 63.4 16.7, 18.8 50.0, 80.0 73.3, 80.0	19.5, 19.5 58.3, 72.7 7.1, - -, -	22.0, 17.0 25.0, 9.1 21.4, 20.0 20.0, 20.0		9.8, - -, - 21.4, - 6.7, -					
*S. canis* *	All LFCA FLEX CAVD	78.6, 74.1 50.0, 100 70.0, - 100, 100	10.7, 25.9 -, - 30.0, 100 -, -					3.6, - 16.7, - -, - -, -			
*E. coli*	All LFCA FLEX CAVD	16.7, 66.7 -, 100 100, - -, -			-, 33.3 -, - -, 100 -, -			16.7, - 100, - -, - -, -	50.0, - -, - -, - 75.0, -	16.7, - -, - -, - 25.0, -	
*E. faecalis*	All LFCA FLEX CAVD					86.7, 80.0 100, 80.0 -, - 33.3, -					13.3, 20.0 -, 20.0 -, - 66.7, -
*P. aeruginosa*	All LFCA FLEX CAVD	7.7, - 33.3, - -, - -, -		7.7, - -, - -, - 20.0, -	15.4, - -, - 40.0, - -, -		7.7, - -, - -, - 20.0,	7.7, - -, - 20.0, - -, -		46.2, 100 66.7, 100 40.0, - 40.0, 100	7.7, - -, - -, - 20.0, -
*Proteus* spp.	All LFCA FLEX CAVD			25.0, - 100, - -, - -, -					75.0, - -, - 100, - 100, -		
*C. auriscanis*	All LFCA FLEX CAVD						-, 20.0 -, - -, - -, 50.0	100, 80.0 100, 100 100, 100 -, 50.0			

Example in bold: 5.3% of *Staphylococcus pseudintermedius* (*S. p.*) misidentifications were classified as *S. aureus* (CAVD, 24 h).

* *Due to a few empty plates, the sum per row is not 100%*.

**Table 6:** Classification of error categories in bacterial identification on chromogenic media by inexperienced veterinary clinicians [based on errors in medicine and medical laboratories (26, 27)].

Error category	Description	Type	Cause	Preventability	Corrective action
Chromogenic medium	Colonies too similar (color and morphology)	Latent (equipment-based)	Test design	Low	Improved or Disease-adjusted chromogenic media
Evaluator (experience- and knowledge-based)	Differences in colonies not recognized	Active – execution (knowledge-based)	Lack of skills and training	Medium	Training Cognitive aids (manuals)
Evaluator (negligence-based)	Sample history not considered	Active – planning (slips/lapses)	Distraction, fatigue, carelessness	High	Checklist Improved working conditions

## 4. Discussion

Skin infections are one of the most common reasons for prescribing antibiotics in veterinary medicine (1). Thus, it is essential to confirm whether the infection is bacterial, what contributes to the diagnostic dilemma of when to prescribe antibiotics (and which), or to wait for antimicrobial susceptibility testing (AST) results (1, 3). Currently, certified laboratories perform most microbiological tests due to the specific knowledge and experience needed. Despite high accuracy, the traditional laboratory-based culture and AST often lack convenience for veterinary clinics regarding turnaround time and costs. For that reason, straightforward point-of-care (POC) tests based on chromogenic media could be a good alternative due to their affordability, speed (~24 h), and in-clinic use if ensuring microbiological safety (10, 12). Chromogenic culture media have been developed over the past 25 years to identify mostly pathogenic or resistant bacteria, like methicillin-resistant *S. aureus* (MRSA), in medicine and food safety (28).

The common chromogenic medium-based POC tests are meant to identify a wide range of pathogens in urinary tract infections (UTI) on a single agar plate. In humans, these tests exhibited high >90% sensitivity and specificity (29, 30). In veterinary studies, only experienced evaluators achieved similar accuracy (20, 21, 23, 24), while beginners correctly recognized 53% (20), 68.7% (21), and, in this study, ~80% samples. The reason for the improved accuracy of inexperienced evaluators in this study could lie in the enrolled pure (not mixed) cultures prepared by a microbiologist. Moreover, we studied pathogens from skin and ears (versus urinary tract in other studies), and the evaluators knew the possible bacterial species in advance, although a limited range of bacteria should not significantly impact overall accuracy due to the inclusion of the majority of skin pathogens (31). However, bacterial species’ prevalence could significantly impact medium performance in practice. For example, veterinarians in areas with higher *S. schleiferi* frequency (32) would have more difficulty identifying a specific pathogen because *S. schleiferi* colonies seemed not to differ enough to be distinguished from other staphylococci (Tables 4, 5).

On all agars, evaluators quickly recognized bacteria growing in distinct colors like green-brown *P. aeruginosa*, blue *E. faecalis*, and orange-brown *Proteus* spp. (Table 4). Colors developed the quickest on the LFCA medium, leading to the highest identification accuracy after 24 h incubation, which did not improve significantly with prolonged 48 h incubation. Rapid colony coloration after 24 h was especially evident for pinkish *S. pseudintermedius* (still being pale on the other two media), which was important to prevent confusion with more reddish *E. coli* colonies (see mistakes in FLEX agar, Table 5). Therefore, LFCA enabled excellent distinction of *S. pseudintermedius*, also from other staphylococci (Table 4). However, one strain of *S. aureus* exhibited bluish color on LFCA and was mistaken for *E. faecalis* (Figure 3). This misidentification happened even though the colonies’ shape was not typical of *E. faecalis*, indicating that beginners seem to rely more on colony color than its morphology. Inexperience could also be a reason why evaluators recognized only ~60% *E. faecalis* samples even after prolonged incubation. Evaluators labeled most misidentified plates as *S. canis*, which colonies exhibited different shades and sizes (Figure 4).

Opposite from LFCA, FLEX agar improved its performance after prolonged 48 h incubation, achieving the highest mean identification accuracy of 86.3% (Table 2). Since Flexicult® Vet is meant for urinary tract infections (UTI), evaluators perfectly recognized typical urinary pathogens such as *E. coli, E. faecalis*, and *Proteus* spp. (Table 4). However, the FLEX was less optimal for skin pathogens, especially after 24 h of incubation. The slow color formation of staphylococcal colonies was probably the reason for suboptimal agar performance. The same problem was also recognized by Guardabassi et al. (20), who recommended incubating Flexicult® Vet longer (48 h instead of 24 h) for *S. pseudintermedius* to develop typical colony colors.

CAVD medium was excellent in identifying *E. coli* due to its distinctive green color (Figure 2). Oppositely, all staphylococci grew in similar colors, including otherwise yellowish *S. aureus*. This was probably the main reason for generally lower CAVD accuracy compared to other media (Table 2). However, CAVD performance was comparable to the other two agars when differentiation between staphylococci was excluded.

Occasional staphylococci species misidentifications do not pose a significant risk for clinical practice and canine patient care (3). Moreover, minimal inhibitory concentration (MIC) and zone diameter breakpoints are the same for AST in the staphylococci genus (33). However, it is important to identify potentially multiple resistant bacteria like methicillin-resistant *S. pseudintermedius* (MRSP) and *S. aureus* (MRSA). In these cases, AST is necessary due to the limited selection of suitable antibiotics like non-beta-lactam fluoroquinolones (enro- or marbofloxacin). Furthermore, resistant staphylococci carry additional hazards due to potential transmission to other animals and humans.

Bacterial intrinsic resistances leading to different antibiotic treatment protocols are another reason for the importance of correct species identification. For example, there were misidentifications between *S. pseudintermedius* and *P. aeruginosa*. The latter, usually growing in distinguishable light green color (Figure 2), often exhibits resistance to multiple classes of antimicrobials like beta-lactams, including penicillins (e.g., amoxicillin) and cephalosporines (cefalexin), fluoroquinolones (enrofloxacin), and aminoglycosides (tobramycin) (34). Furthermore, misidentifying *S. pseudintermedius* for *S. canis* could have led to a serious mistake in the presence of MRSP. Oppositely, *S. canis* demands only a straightforward protocol with penicillins (35), and labeling the species as MRSP would cause additional AST-related costs. We think that the inexperience of the evaluators was the main contributing factor to these errors because *S. canis* exhibits evidently smaller colonies on all three enrolled media. As we demonstrated in our previous study, the experience helped to consider all colony characteristics leading to better bacterial identification (21).

Our study additionally revealed several misidentifications between cocci and rods (Table 5), like *S. pseudintermedius* vs. *E. coli* and *E. faecalis* vs. *C. auriscanis*. Since information on bacterial shape was known in advance (from presumed cytology), these errors can only be attributed to the evaluator’s negligence. Moreover, misidentifying feline *S. felis* for canine *S. pseudintermedius* was surprising because the origin of the analyzed isolates was known to evaluators, and we informed them that either species occurs only in one animal (i.e., we did not retrieve any *S. pseudintermedius* in cats). The negligence-related misidentifications above accounted for 8.9–11.7% of all mistakes (Tables 4, 5).

Most errors revealed in this study can be categorized (26, 27) and related to (i) chromogenic medium, (ii) evaluator (in)experience, and (iii) negligence (Table 6). We had initially expected that media-caused errors would be dominant, causing inter-rater variability to be smaller than disagreement with the gold standard (Table 3). Although evaluators relied strongly on colors and many errors happened due to similar colony shades (e.g., staphylococci), all agreements were comparable, indicating various errors evaluators had made. It seemed that each evaluator responded individually when presented with uncertainty. Unfortunately, the study design did not allow us to estimate the proportions of error related to medium design or evaluator (in)experience. Previous studies showed that, in some instances, an expert familiar with the particular medium perfectly identified bacterial species (20). Because presuming ideal bacterial prevalence for specific agar and perfection reached by all experts is not realistic (21), we suggest further agar improvements. On the one hand, advances in agar design can focus on maximizing general performance in identifying veterinary bacteria, e.g., by optimizing the type and concentration of chromogenic enzyme substrates added. Alternatively, and more reasonably, media could be adjusted for a specific organ system like skin, focusing on better staphylococci differentiation.

Additional personnel training, evaluation flowchart, and imaging or other visual support could prevent identification errors in recognizing less noticeable or not color-related differences in bacterial colonies. Interactions with clinical microbiologists to discuss the bacterial growth on agar plates in real time would also help clinicians interpret unfamiliar cases and provide important educational processes. Furthermore, this study showed that around 10% of errors could not be explained by the medium design and evaluator experience. We classified them as negligence-related slips and lapses as a result of evaluator distraction or fatigue. Due to many agar plates (i.e., 30–90) being evaluated in a single session, the study definitely provoked a significant load on the enrolled evaluators, causing stress, distraction, and fatigue. These emotional and mental conditions are often present in bustling veterinary clinics driving veterinarians to perform various laboratory tests in a hurry or at the end of the shift. However, establishing suitable working conditions and checklists, reminding the evaluator to consider sample history, could significantly improve bacterial identification on the chromogenic media.

## 5. Conclusion

Translating chromogenic media into veterinary clinics can significantly advance current in-clinic diagnostics of canine and feline pyodermas and external otitises. Cytology reveals only the presence and shape of bacteria (cocci and rods), while chromogenic agars enable the identification of skin pathogens with around 80% accuracy. The extra information can help clinicians choose treatment protocols or opt for standard antimicrobial susceptibility testing (AST). Knowing bacterial species is particularly useful for initial, non-complicated infections (assuming local epidemiological data is known).

Various general chromogenic agars (including our CAVD) exhibit similar performance accuracies and seem suitable for introduction into clinics. On all media, inexperienced evaluators easily identified unique-colored colonies of bacteria like *P. aeruginosa*, *E. faecalis*, and *Proteus* spp. Challenges arose when the morphology (colony size) was essential or bacteria grew in similar shades (staphylococci). Around 10% of errors were slips related to not considering sample history. To improve bacterial identification, media could be improved to serve specific purposes or organ systems like distinguishing between skin staphylococci species. Additional personnel training, evaluation help by visuals, flowcharts, checklists, and, if necessary, microbiologists can all further improve identification accuracy.

Future research should add real, often mixed bacterial skin cultures prepared by clinicians directly from clinical samples. We shall also test if chromogenic media with added antibiotics could be applied for AST in skin infections. We do not think that in-clinic POC tests are meant to replace standard AST in microbiological laboratories but to fill a gap between currently dominant empirical antibiotic treatments and rarely applied standard AST.

## Data availability statement

The raw data supporting the conclusions of this article will be made available by the authors, without undue reservation.

## Ethics statement

The animal study was reviewed and approved by the ethics committee, Ministry of Agriculture, Forestry and Food, Republic of Slovenia. Written informed consent was obtained from the owners for the participation of their animals in this study.

## Author contributions

MA and BC ideated, designed, and performed the experiments. JI designed the CAVD agar and provided guidelines on its use in veterinary settings. BC performed the statistics. All authors contributed to the article and approved the submitted version.

## Funding

This research was funded by the European Society of Veterinary Dermatology (ESVD, Minor grant), the Latvian State Education Development Agency (1.1.1.2/VIAA/3/19/455), and the Slovenian Ministry of Economic Development and Technology under the European Regional Development Fund (Eureka E! 13509).

## Conflict of interest

JI, GF and US are employed by Biosynth AG.

The authors conducted the experimental design, experiments, and data analysis independently. Biosynth AG (Staad, Switzerland) provided the chromogenic substrates and the inducer compounds for the CAVD agar preparation at no cost. Aldol® reagents are proprietary patent-protected products of Biosynth AG. Liofilchem Chromatic™ MH and Flexicult® Vet Scandinavia were purchased from the official distributors.

## Publisher’s note

All claims expressed in this article are solely those of the authors and do not necessarily represent those of their affiliated organizations, or those of the publisher, the editors and the reviewers. Any product that may be evaluated in this article, or claim that may be made by its manufacturer, is not guaranteed or endorsed by the publisher.

## Figures and Tables

**Table:** 

AST	Antimicrobial susceptibility testing
C.	*Corynebacterium*
CAVD	Custom-made chromogenic medium based on the Mueller Hinton II agar
CFU	Colony-forming units
E.	*Escherichia*
FLEX	Flexicult Vet Scandinavia
IRR	Inter-rater reliability
LFCA	Liofilchem Chromatic MH
MALDI-TOF	Matrix-assisted laser desorption/ionization time-of-flight
MIC	Minimal inhibitory concentration
MRSA	Methicillin-resistant Staphylococcus aureus
MRSP	Methicillin-resistant Staphylococcus pseudintermedius
P.	*Pseudomonas*
POC	Point-of-care tests
S.	*Staphylococcus*, or *Streptococcus* for *S. canis*
UTI	Urinary tract infections
